# WHITE BOX: LOW COST BOX FOR LAPAROSCOPIC TRAINING

**DOI:** 10.1590/S0102-67202015000300015

**Published:** 2015

**Authors:** João Maximiliano Pedron MARTINS, Roberto Vanin Pinto RIBEIRO, Leandro Totti CAVAZZOLA

**Affiliations:** Clinics Hospital of Porto Alegre, Porto Alegre, RS, Brazil

**Keywords:** Laparoscopy, Minimally invasive surgical procedures, Medical education, Surgery

## Abstract

**Background:**

Laparoscopic surgery is a reality in almost all surgical centers. Although with initial greater technical difficulty for surgeons, the rapid return to activities, less postoperative pain and higher quality aesthetic stimulates surgeons to evolve technically in this area. However, unlike open surgery where learning opportunities are more accessible, the laparoscopic training represents a challenge in surgeon formation.

**Aim:**

To present a low cost model for laparoscopic training box.

**Methods:**

This model is based in easily accessible materials; the equipment can be easily found based on chrome mini jet and passes rubber thread and a webcam attached to an aluminum handle.

**Results:**

It can be finalized in two days costing R$ 280,00 (US$ 90).

**Conclusion:**

It is possible to stimulate a larger number of surgeons to have self training in laparoscopy at low cost seeking to improve their surgical skills outside the operating room.

## Abstract (pt)

**Racional:**

A cirurgia laparoscópica já é realidade em praticamente todos os centros cirúrgicos. Muito embora haja maior dificuldade técnica, o rápido retorno às atividades, menor dor pós-operatória e maior qualidade estética estimulam os cirurgiões a evoluírem tecnicamente nesta área. Porém, ao contrário das operações abertas onde as oportunidades de aprendizagem são mais acessíveis, o treinamento laparoscópico acaba sendo desafio ao longo da formação de um cirurgião.

**Objetivo:**

Apresentar um modelo de caixa de treinamento laparoscópico de baixo custo.

**Métodos:**

Foram utilizados materiais de fácil acesso, mini jato cromado, passa fios de borracha e uma webcam acoplada a um cabo de alumínio.

**Resultados:**

A white box foi produzida em dois dias com custo de R$ 280,00.

**Conclusão:**

É possível estimular o treinamento de maior número de cirurgiões na formação laparoscópica a baixo custo e buscar aperfeiçoamento de suas habilidades cirúrgicas fora do centro cirúrgico.

## INTRODUCTION

Laparoscopic surgery is already a reality in virtually all surgical centers with precise indications and good results in almost all specialties. Over the past few years the number of procedures done laparoscopically is growing. And, although there is a greater initial technical difficulty, the rapid return to activities, less postoperative pain and better aesthetic quality stimulate surgeons to technically evolve in this area 1 .

Currently medical students already have contact with many laparoscopic operations from the beginning of their training, which leads to the need to acquire technical skills both for open and laparoscopic procedures. However, unlike conventional surgeries, where learning opportunities are more accessible in the surgical field, laparoscopic training ends up being a challenge over a surgeon's education because of the difficulty of access to materials and high costs of training. Laparoscopic operations require a greater learning curve, especially due to the difficulty in adapting to the bidimensional vision - called the fulcrum effect -, but also because of the new instruments that need to be mastered 1 . Several studies have shown that training with simulators can greatly increase surgical abilities 3 6 7 8 ; however, this alternative is inaccessible to many centers due to high cost and complexity of creating a laparoscopic training lab with proper equipment and supervision within university hospitals. Thus, recent studies are beginning to suggest that simple acrylic or wooden boxes adapted to webcams can help surgeons to develop their technical abilities^6^, with significantly lower cost and unlimited access to the equipment.

The objective of this study was to present a low-budget laparoscopic training box model.

## METHODS

**FIGURE 1:**
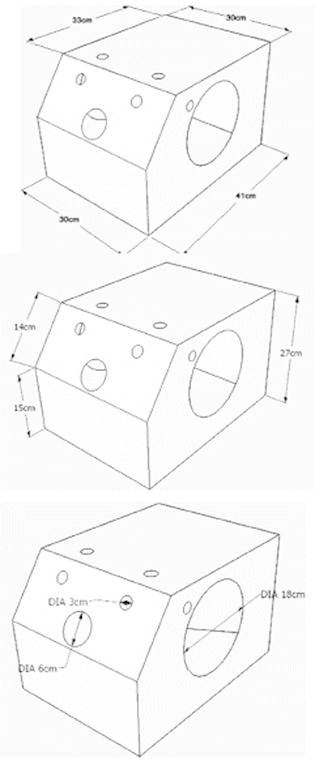
Format and dimensions of the laparoscopic training model

Resistant, lightweight and low-cost material is required to develop the appropriate box. To do this, MDF can be used with a thickness of 5 mm. The dimensions of the box are shown in Figure 1 .

**FIGURE 2:**
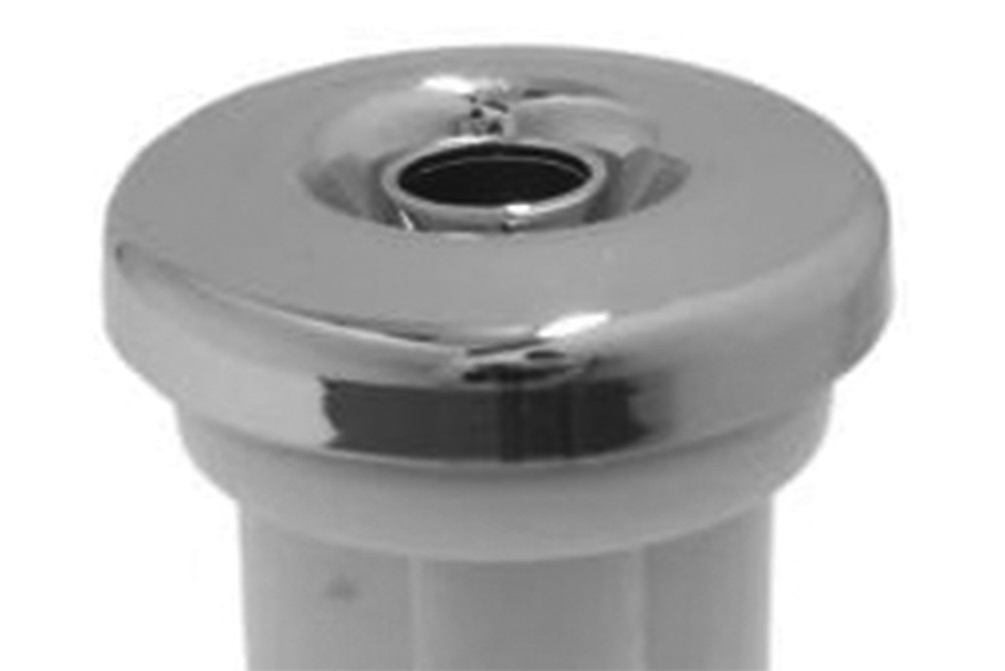
Mini chromed jet

To approach the sensation of reality in camera manipulation, a device called "mini chrome jet" must be coupled in the box, this is easily found for sale ( Figure 2 ). Through it, can be given mobility to the camera as in the actual operations. This device allows an assistant to move the camera while the surgeon handles the operating instruments (which involves the simultaneous training of two students), and also allows the individual training because when you release the camera the device is stabilized in the same location.

**FIGURE 3:**
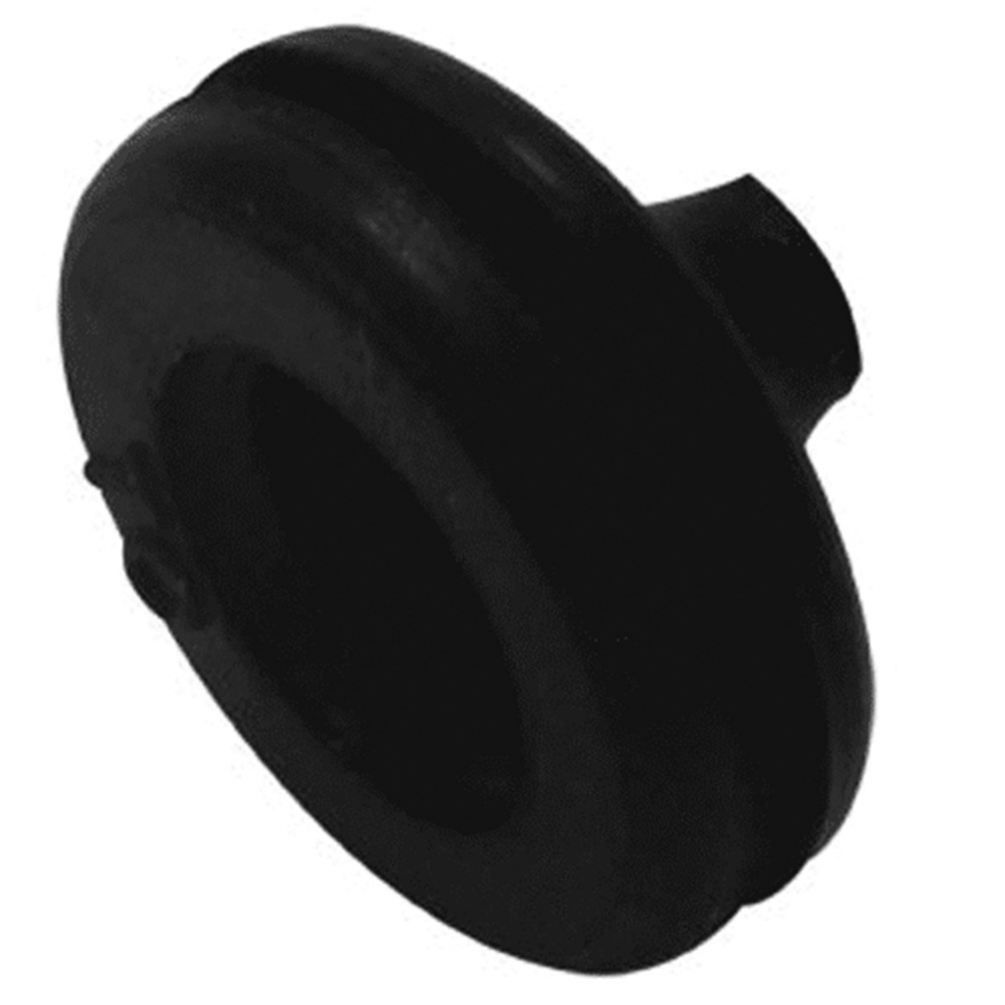
Rubber thread passer

To simulate the adhesion of the skin with the instruments, rather than the traditional trocar used in the procedures, "rubber thread passer" should be attached ( Figure 3 ), which allows mobility while providing a similar resistance to human skin.

**FIGURE 4:**
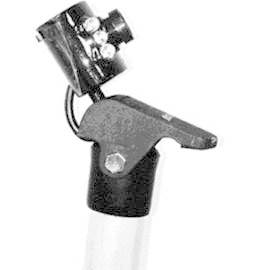
Support and webcam

The camera is a webcam attached to a 3 mm aluminum cable coated with a transparent hose allowing the necessary grip when in contact with the "mini chrome jet" ( Figure 4 ).

**FIGURE 5:**
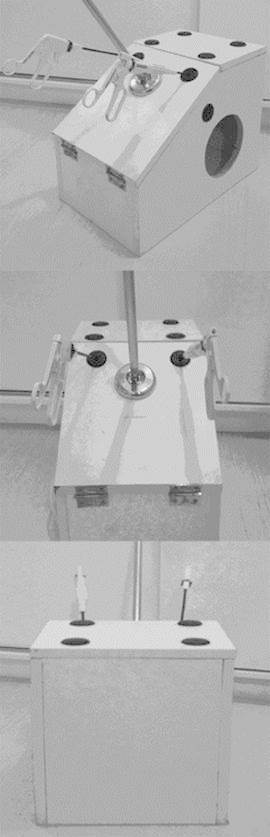
White box ready for use requiring the addition of a monitor to expose the image coming from the webcam box

## RESULTS

For the making of this model ( Figure 5 ) approximately R$ 250.00 were spent and it took two days of work.

## DISCUSSION

Several studies have proven that training with the use of virtual reality simulators or boxes is effective to improve the skills of surgeons and decrease perioperative complications^4^. In addition, the training of basic laparoscopic techniques directly in the operating room and on patients is not cost-effective and is potentially hazardous 1 7 .

Another important fact to be emphasized is that it has been shown that the use of training boxes using webcams are as effective as the boxes using high-definition laparoscopic cameras for training laparoscopic skills 1 2 . It has also been shown that the use of low-budget boxes for household training is equally or more effective than more sophisticated simulators used in large training centers, because they provide the studied subjects more time and freedom to practice 5 . Excellent imported boxes are available in the market (3D-MED, large T5), but their cost is around US$ 2,700.00, limiting their acquisition to individual and private training.

This article describes one of the ways to set up low-budget training box that uses a webcam for viewing. Previous papers have described other forms to mount low-cost training boxes 1 . In this study, however, is demonstrated a suitable model for the practice of laparoscopic techniques. Besides having a more rigid and durable structure, the white-box has an angled shape that helps mimic the circumference of the abdomen. Also, this box uses the "mini jet chrome", which allows the trainee to set the camera at a specific point with no need for other individuals to aid in the training, but, at the same time, it allows two persons to train at the same time when so desired.

## CONCLUSIONS

It is possible to stimulate the training of more students and surgeons in laparoscopic surgery at a low-cost and also seek improvement of their surgical skills outside the operating room.
